# A phase II study for metabolic *in vivo *response monitoring with sequential ^18^FDG-PET-CT during treatment with the EGFR-monoclonal-antibody cetuximab in metastatic colorectal cancer: the Heidelberg REMOTUX trial

**DOI:** 10.1186/1471-2407-12-108

**Published:** 2012-03-22

**Authors:** Anne Katrin Berger, Carl von Gall, Ulrich Abel, Stefan Delorme, Matthias Kloor, Jennifer Ose, Tim Frederik Weber, Annika Stange, Georg Martin Haag, Uwe Haberkorn, Florian Lordick, Dirk Jäger

**Affiliations:** 1 National Center for Tumor Diseases (NCT), University Medical Center Heidelberg, Heidelberg, Germany; 2 Department of Nuclear Medicine, University Hospital Erlangen, Erlangen, Germany; 3 Department of Radiology, German Cancer Research Center, Heidelberg, Germany; 4 Department of Applied Tumor Biology, University Medical Center Heidelberg, Heidelberg, Germany; 5 Department of Diagnostic and Interventional Radiology, University Medical Center Heidelberg, Heidelberg, Germany; 6 Department of Nuclear Medicine, University Medical Center Heidelberg, Heidelberg, Germany; 7 Department of Oncology and Hematology, Hospital of Braunschweig, Braunschweig, Germany; 8 Institute of Medical Biometry and Informatics, University Medical Center of Heidelberg, Heidelberg, Germany

**Keywords:** Colorectal cancer, Metastases, Cetuximab, Metabolic imaging, ^18^F-FDG PET CT

## Abstract

**Background:**

The epidermal growth factor receptor monoclonal antibody cetuximab has proven activity in metastatic colorectal cancer. To date, the mechanisms of action are not completely understood. Especially the impact on tumor glucose metabolism, or tumor vascularization remains largely unclear. The understanding of mechanisms such as early changes in tumor metabolism is of clinical importance since there may be a substantial influence on choice and sequence of drug combinations. Early signals of response to cetuximab may prove useful to identify patients having a relevant clinical treatment benefit. The objective of this trial is to evaluate the predictive relevance of the relative change in ^18 ^F-Fluorodeoxyglucose tumor uptake for early clinical response during short-term single agent treatment with cetuximab. Early clinical response will be routinely measured according to the response evaluation criteria in solid tumors. Accompanying research includes cytokine immune monitoring and analysis of tumor proteins and tumor genes.

**Methods/design:**

The REMOTUX trial is an investigator-initiated, prospective, open-label, single-arm, single-center early exploratory predictive study. The first ^18 ^F-FDG PET-CT is conducted at baseline followed by the run-in phase with cetuximab at days 1 and 8. At day 14, the second ^18 ^F-FDG PET-CT is performed. Subsequently, patients are treated according to the Folfiri-cetuximab regimen as an active and approved first-line regimen for metastatic colorectal carcinoma. At day 56, clinical response is evaluated with a CT-scan compared to the baseline analysis. Tracer uptake is assessed using standardized uptake values (SUVs). The main hypothesis to be tested in the primary analysis is whether or not the relative change in the SUV from baseline to day 14 has any predictive relevance for early clinical response determined at day 56. Patients are followed until death from any cause or until 24 months after the last patient has ended trial treatment.

**Discussion:**

The aim of this trial is to evaluate metabolic changes in metastatic colorectal cancer during short-term single agent treatment with cetuximab and to analyse their potential of predicting early clinical response. This could be helpful to answer the question if early identification of patients not responding to cetuximab is possible.

**Trial registration:**

ClinicalTrials.gov NCT200811021020; EudraCT 200901327923

## Background

Colorectal cancer is one of the three most common types of cancer in men and in women. It is estimated that about 1.2 million new cases were diagnosed worldwide in 2008 and approximately 609.000 deaths occurred [1]. The 5-year overall survival rate for patients with metastatic disease in the western world has increased during the last decade and is nowadays reported to reach about 10% [1]. The median overall survival for patients treated with active combination chemotherapies and monoclonal antibodies (mABs) is nowadays in excess of two years [2].

The epidermal growth factor receptor (EGFR) mAB cetuximab has proven activity in metastatic colorectal cancer. In combination with an irinotecan-containing cytotoxic regimen (Folfiri), cetuximab significantly increases progression free survivial (PFS) in first-line therapy compared to the Folfiri regimen alone [3]. In patients with metastatic colorectal cancer that no longer respond to previous chemotherapy for advanced disease, cetuximab significantly improves overall survival (OS) and PFS compared to best supportive care alone [4]. More recently, it has been demonstrated that efficacy of cetuximab is significantly associated with a wild-type KRAS status [5,6]. Cetuximab is approved by the European Medicines Agency (EMEA) for the treatment of patients with EGFR expressing, KRAS wild-type metastatic colorectal cancer (in combination with chemotherapy as well as single-agent-therapy in patients who have failed oxaliplatin- and irinotecan-based therapy and who are intolerant to irinotecan). Nevertheless, there is still a group of patients with wild-type KRAS status that does not benefit from treatment with cetuximab, and additional mechanisms of resistance are assumed. Despite all advances, it is yet not possible to identify patients who will respond to cetuximab treatment upfront. Given the substantial therapy costs and the considerable rate of side effects (especially skin toxicity), improved strategies for identifying responders are needed. Patients who are responsive to cetuximab treatment may gain a tremendous benefit if combinations of chemotherapy and cetuximab are given in the first-line situation, because a switch from primarily palliative chemotherapeutic treatment to a curative surgical approach („conversion therapy") seems to be more prevalent in patients who are responsive to induction treatment [7].

To date, the influence of cetuximab on tumor glucose metabolism, tumor vascularization and angiogenesis remains largely unclear. The understanding of these processes such as early changes in tumor glucose uptake or changes in blood flow parameters is of utmost clinical interest since early signals of response to cetuximab may prove useful to identify those patients who have a relevant clinical benefit from treatment. In general, the role of metabolic imaging and early response assessment to anticancer therapy is one of oncology's key questions today. The implications for therapeutic management and treatment strategies, especially in patients with gastrointestinal cancer, are a point of intensive discussion [8]. For example, in locally advanced adenocarcinoma of the esophagogastric junction, the MUNICON trial showed the value of early metabolic response evaluation and demonstrated the feasibility of a PET-guided treatment algorithm in clinical practice [9]. The potential of sequential ^18 ^F-FDG PET (18 F-Fluorodeoxyglucose positron emission tomography) in patients with adenocarcinoma of the esophagogastric junction under salvage radiotherapy is actually investigated by the HICON trial [10]. Referring to ^18 ^F-FDG PET, changes of the standardized uptake value (SUV) rather than the absolute SUV values are considered to be the most reliable parameter for therapeutic response assessment [11].

The primary objective of the REMOTUX trial (*in vivo response monitoring of treatment with the EGFR-mAB cetuximab in metastatic colorectal cancer*) is to evaluate the predictive relevance of the change in ^18 ^F-FDG tumor uptake during short-term run-in treatment with cetuximab for the early clinical tumor response, determined according to the response evaluation criteria in solid tumors (RECIST) [12] after sequential Folfiri-cetuximab therapy. Antivascular and/or antiangiogeneic effects of cetuximab by contrast-enhanced ultrasound will also be assessed in the secondary analysis. An accompanying translational research programm analyses EGFR dependent signaling pathways and immunological parameters influenced by cetuximab.

## Methods/design

### Ethical and legal considerations

The REMOTUX study protocol, the patient information and informed consent sheets for study participation and additional translational research were approved by the local ethics committee at the University of Heidelberg. Additionally, the study was authorised by the Paul-Ehrlich-Institut (as an agency of the German Federal Ministry of Health) and by the German Federal Authorities for Radiation Protection (Bundesamt für Strahlenschutz) according to federal law. The trial is registered at the ClinicalTrials.gov protocol registration system (identification code NCT200811021020) and the EudraCT register (identification code 200901327923). All patients provide written informed consent before study inclusion. A patient may be withdrawn from the trial treatment at any time at his own request

### Study design and treatment schedule

The REMOTUX trial is designed and conducted at the National Center for Tumor disease (NCT) at the University Medical Center Heidelberg. It is an investigator-initiated, prospective, open-label, single-arm, and single-center early exploratory predictive study.

The first ^18 ^F-FDG PET-CT (and liver ultrasound, if available), is conducted at baseline followed by the run-in treatment phase with cetuximab therapy at days 1 and 8 (400 mg/m^2 ^bsa and 250 mg mg/m^2 ^bsa respectively). At day 14, the second ^18 ^F-FDG PET-CT (and liver ultrasound, if available) is performed. Subsequently, patients are treated according to the Folfiri-cetuximab regimen as an active and approved first-line regimen for metastatic colorectal carcinoma. According to the clinical standards at the NCT Heidelberg, the Folfiri-cetuximab regimen is recommended to follow the schedule of Table 1. At day 56, the early clinical response is evaluated with a routine CT-scan in comparison to the baseline analysis. Depending on the clinical response, treatment will be continued according to the choice of the responsible physician. It is recommended that, in case of response, treatment will be continued with cetuximab and the Folfiri regimen until disease progression or patients are unable to tolerate the therapy. Patients are followed-up every 3 months. Patients are followed until death from any cause or until 24 months after the last patient has ended trial treatment. A flowchart is given in Table 2.

**Table 1:** Folfiri-Cetuximab-regimen, to be repeated every 2 weeks

Chemotherapeutic agent	**Dosage (mg/m**^**2**^**)**	Treatment day
Cetuximab	250 *	1, 8
	* 400 at first application	
Irinotecan	180	1
Folinic Acid	400	1
5Fluorouracil (bolus)	400	1
5Fluorouracil (46 hours)	2400	1

**Table 2:** Flowchart (diagnostic procedures and run-in treatment)

Baseline	Day 1	Day 8	Day 14		Day 56
PET-CT	Cetuximab	Cetuximab	PET-CT	Folfiri-Cetuximab	CT

### Diagnostic procedures

#### ^18^F-FDG PET-CT

For ^18 ^F-FDG PET-CT examination, patients are required to fast at least 6 hours prior to the application. Diabetes mellitus should be treated properly in advance but the use of insulin is not allowed. The radiopharmaceutical ^18 ^F-FDG is given intravenously (i.v.). Administered activity is estimated between 3.5 and 5 MBq/kg of bodyweight. If there are no contraindications, 20 to 40 mg butylscopolaminiumbromide will be administered i.v. to reduce bowel movement and therefore the physiological glucose uptake. Bowel distension using water and 10 ml of 10% mannitol will be performed additionally for oral contrast enhancement. After identification of the tumor using a ultra low dose scout scan, the tracer substance will be administered intravenously and a dynamic acquisition over 60 minutes will start, followed by a whole body scan from head to upper femur. In addition, a diagnostic routine CT using intravenous contrast enhancement will be performed after PET acquisition at baseline. SUVs will be used for quantitative ^18 ^F-FDG PET-CT analysis to evaluate the changes of glucose metabolism. Using baseline and follow-up ^18 ^F-FDG PET-CT and the related SUVs, the predictive relevance of the relative changes in SUV for early clinical response will be evaluated. PET/CT scans will be performed on a SIEMENS PET/CT Biograph 6 (SIEMENS Healthcare, Erlangen Germany) with an axial field of view of 15,4 cm in 3D mode. All images will be reconstructed using OSEM 2D, four iterations and eight subsets. All image data set is normalized for the injected dose and thepatients body weight. This results in parametric imaging using widely accepted standardized uptake value (SUV) on the basis of the formula "SUV = tissue concentration (Bq/g)/(injected dose (Bq)/body weight (g))".

For quantitative evaluation, an automated volume of interest (VOI) derived from generated regions of interest (ROI) using the auto3D function within the SYNGO Software (Siemens Healthcare Erlangen, Germany) will be placed over the tumor. In the second PET scan, the region of interest will be placed at the same position as in the baseline PET as a reference of initial maximal uptake.

#### Contrast-enhanced ultrasound (CEUS)

For contrast-enhanced ultrasound, the i.v. application of 2.4 ml SonoVue^® ^(sulfurhexafluoride-microbubbles) is necessary. SonoVue^® ^is approved for ultrasound examination to improve echogenicity of blood and vessel structures from time-intensity curves obtained during constant infusion, and using dedicated software for the analysis of replenishment kinetics. Furthermore, methods have been developed at the German Cancer Research Center (Deutsches Krebsforschungszentrum, DKFZ) which directly measure relative blood volume and perfusion.

#### Collection and processing of samples

If special written informed consent for translational research is available, 20 ml of venous blood will be taken at baseline and on day 14 for cytokine immune monitoring during the study. Tumor protein and tumor gene expression analyses will be performed on the existing pre-therapeutic biopsy specimen, if available.

### Study objectives

The primary objective of this study is to evaluate the predictive relevance of relative changes in SUV measured with ^18 ^F-FDG PET-CT during short-term run-in treatment with the EGFR-mAB cetuximab for the early clinical response (as defined by RECIST). The study is neither aimed at demonstrating an increase in the early clinical response rate compared to an assumed rate of a conventional treatment, nor to show that the early clinical response rate of the study treatment is higher than a given „uninteresting" rate. The trial's secondary objectives are the duration of PFS and OS as well as the influence of changes in individual SUV and early clinical response on PFS and OS, respectively. Additionally, for patients with liver metastases there is an assessment of antivascular/antiangiogenic effects of cetuximab by contrast-enhanced ultrasound on baseline and day 14. The clinical trial includes an accompanying research program involving collection of biological samples for pseudonymized analyses. These comprise sequential serum protein marker assessments (like multiplex cytokine immune monitoring) as well as baseline analysis of tumor proteins and tumor genes. Patients can participate in this study even if they choose not to participate in this translational research program.

### Trial population

Patients with untreated metastatic colorectal cancer can participate in the REMOTUX trial when the eligibility criteria are met. Those criteria include histologically confirmed metastatic colorectal cancer with a KRAS-wildtype status of the tumor and no history of previous therapy with an EGFR-targeting agent or previous chemotherapy for advanced disease. Patients must have an adequate hematologic, renal and hepatic function and must be able to undergo chemotherapy according to the Folfiri regimen. Patients with an ECOG-performance status less than 1, CNS metastases or an uncontrolled diabetes mellitus can not participate in the study. For imaging, there must be at least one measurable tumor lesion with a diameter no smaller than 1.0 cm detected by CT, MRI or ultrasound and for contrast-enhanced ultrasound the liver metastases must not be smaller than 2.0 cm. The detailed criteria are shown in Table 3.

**Table 3:** Detailed inclusion and exclusion criteria

Inclusion criteria	Exclusion criteria
• Histologically confirmed metastatic colorectal cancer • KRAS-wildtype status of the tumor • No history of therapy with an EGFR targeting agent • No history of previous chemotherapy for advanced disease • Measurable tumor lesion with a diameter no smaller than 1.0 cm detected by CT, MRIor ultrasound • For contrast-enhanced ultrasound: metastases no smaller than 2.0 cm detected by ultrasound • ECOG-performance status 0 or 1 or Karnofsky performance scale min. 70% • Life expectancy > 12 weeks • Age ≥ 18 years • Adequate hematologic, renal and hepatic function • Ability of the patient to understand the character and individual consequences of this clinical trial • Written informed consent (must be available before enrolment in the trial) • For women and men with childbearing potential adequate double barrier contraception, for women: negative pregnancy test	• Any contraindications for chemotherapy according to the Folfiri regimen • Non-curatively treated malignancy within the last 5 years • Uncontrolled or insulin-dependant diabetes mellitus • Evidence of CNS metastases • Uncontrolled infection • Significant cardiac disease (unstable angina pectoris or cardiac symptoms according to NYHA classification III or IV) • Active serious illness which renders the patient unsuitable for study entry or multiple blood sampling • Pregnancy and lactation • History of hypersensitivity to cetuximab or to any drug with similar chemical structure or to any excipient present in the pharmaceutical form of the investigational medicinal product •Participation in other clinical trials or observation period of competing trials, respectively

### Statistical considerations

#### Study hypothesis

The main hypothesis to be tested in the primary analysis is whether or not the relative change

(ΔSUV = 100⋅(SUV_Baseline_-SUV_d14_)/SUV_Baseline_) in the SUV from baseline to day 14 has any predictive relevance for early clinical response determined at day 56. This hypothesis is tested by comparing the groups of early clinical responders and nonresponders with respect to the quantitative variable ΔSUV using a Wilcoxon rank sum test (2-sided test, α = 5%)

#### Sample size calculation

The trial will include 35 patients who are evaluable for the primary analysis. The sample size/power calculations were based on the Wilcoxon rank sum test (two-sided testing; α = 5%) in the form proposed by Noether [13] and implemented in the software package nquery advisor 6.01 [Statistical Solutions, Inc.: nquery advisor^® ^6.01. Saugus, MA (2005)]. This form of the power calculation assumes that the alternative hypothesis is expressed as a probability P(Y > X), which, in our context, is the probability that a random responder has a higher value of ΔSUV than a random nonresponder. Note that the probability P(Y > X) is identical to the AUC of the population ROC of ΔSUV with respect to the binary response. It should be observed that the group sizes of this test cannot be determined in advance. Rather, the number of responders follows a binomial distribution with n = number of evaluable patients, and binomial probability r = probability of response. Therefore, the power of the test is a weighted sum of the values of the power calculated for each possible constellation of group sizes of responders and nonresponders, the weights being the binomial probabilities that a particular constellation arises. Assuming that the values of ΔSUV in the groups of clinical responders and nonresponders, resp., are represented by independent normally distributed variables with equal variances, and assuming that the true early clinical response rate of r is in the range 40% to 60%, the projected sample size of 35 evaluable patients is sufficient to detect an AUC of ΔSUV (calculated with respect to response) of 0.8 with power 84.1% (the maximum power of 85.7% being attained for r = 50%). It should be noted, however, that the true clinical response rate r is unknown. For r = 65% (instead of r = 60%) the estimated power for an AUC = 0.8 drops to 81.9%. Therefore, the slight overpowering if r is in the assumed range of values (40% to 60%) appears justified. Assuming that about 10% of the enrolled patients are not evaluable for the primary analysis, it is expected that the total number of patients to be enrolled in the study is 39. It is noteworthy that, under the assumptions specified above, 35 evaluable patients are also sufficient for detecting with power > 80% an AUC of 0.8 in the exploratory analysis of the main end point by means of a univariate logistic regression analysis (calculations based on 10000 computer simulations runs).

#### Statistical analysis

The null hypothesis that ΔSUV has no predictive power for early response will be tested using the Wilcoxon rank sum test (2-sided test, α = 5%). The null hypothesis of this test is that the distributions of ΔSUV in the groups of early clinical responders and nonresponders are identical. The AUC of ΔSUV with respect to the response status will be calculated along with bias-corrected and accelerated bootstrap 95% confidence intervals, as described by Zhou [14]. If, based on the results, a cut-off for predicting response is selected post hoc, then using the bootstrap (and with the same criterion as used in the selection) bias-corrected indices of accuracy will be estimated from the data. Logistic regression models will be used to further explore the relationship between ΔSUV and early clinical response, and, in particular, whether any confounder for the relationship between ΔSUV and early clinical response can be determined. The model assumption of a linear relationship between ΔSUV and the logit of endpoint will be explored by visual inspection. Standard methods for right-censored data will be used for analyzing PFS and OS. The Cox Regression model will be used to examine the influence of ΔSUV and of early clinical response on PFS and OS. For the evaluation of the predictive value of ΔSUV for clinical tumor response all patients will be analyzed for whom the variable ΔSUV as well as the response measurement is available. The assessment of safety will be based mainly on the frequency of adverse events and on the number of laboratory values that fall outside of pre-determined ranges and/or show prominent worsening from baseline during the study phase. Missing values will not be replaced or imputed

### Safety aspects and adverse events (AEs)

Due to the use of a safe and well known therapeutic regimen, there are only few AEs to be expected. Skin toxicity ≤ grade 3 caused by cetuximab will not be reported as an AE. In this trial, all AEs that occur after the patient has signed the informed consent document will be documented. All patients who have AEs, whether considered associated with the use of the trial medication or not, are monitored to determine the outcome. The clinical course of the AE will be followed-up until resolution or normalization of changed laboratory parameters or until it has changed to a stable condition.

### Current status

At the time of writing we have included 7 patients. The recruitment period is expected to last 12 more months. Evaluation of the clinical data will be done within three months after inclusion of the last patients. There is no planned interim analysis.

## Discussion

Given the increasing costs of modern oncologic treatment [15] and the possible toxicities of anticancer drugs, refinements to the use of modern therapeutic agents and therapy strategies are essential to improve oncologic care. For the use of cetuximab, it is already known that effectiveness is restricted to patients with a wild-type KRAS status. Still, a subgroup of these patients does not respond to cetuximab therapy and additional methods for identifying responders in advance are requested. ^18 ^F-FDG PET-CT has demonstrated its value in the early assessment of metabolic response in localized cancer of esophagogastric junction during neoadjuvant combination chemotherapy. Additionally, it was demonstrated to predict clinical outcome in advanced gastric cancer during combination therapy with Folfiri and cetuximab [16] but tumor metabolism has not been analyzed in terms of single agent treatment with an EGFR mAB.

Our study was designed to evaluate metabolic response during run-in single agent therapy with cetuximab in correlation with clinical response. To our knowledge, this is the first analysis concerning the question of changes in tumor glucose metabolism and tumor vascularization under single-agent cetuximab in solid tumors. If there are measureable metabolic changes, their correlation with clinical response will be most interesting. In case of positive correlation, further studies could answer the question if early selection of KRAS- wildtype patients not responding to cetuximab is possible to avoid unneccessary toxic and cost-intensive treatment.

## Competing interests

The authors declare that this study is an investigator-initiated trial, funded by Merck Pharma GmbH. The contract between the financial sponsor and the University of Heidelberg leaves the full responsibility for the scientific work, the management of data, and for analysis and publication to the investigators.

## Authors' contributions

AKB, UA, JO, AS, UH, FL and DJ participated in protocol and study design. AKB, CG, SD, and TFW participate in trial conduction. AKB, AS, GMH and DJ participate in patient recruitment. UA is responsible for statistical planning of the trial. MK carries out translational research. AKB wrote the manuscript. All authors read and approved the final manuscript

## Pre-publication history

The pre-publication history for this paper can be accessed here:

http://www.biomedcentral.com/1471-2407/12/108/prepub
